# Excitons make quantum leap in just 2.5 femtoseconds

**DOI:** 10.1038/s41377-026-02439-7

**Published:** 2026-08-21

**Authors:** O. Neufeld

**Affiliations:** Technion – Israel Institute of Technology, Schulich Faculty of Chemistry, Haifa, 3200003 Israel

**Keywords:** Ultrafast photonics, Electronics, photonics and device physics, Solar energy and photovoltaic technology, High-harmonic generation

## Abstract

Light irradiation in two-dimensional materials is well-known to excite tightly-bound many-body excitonic states that dominate optical responses. Recent theory analysis under intense laser conditions identifies the mechanisms and timescales through which excitons form, evolve, and dissociate. These findings open new opportunities for sensing and optically controlling many-body physics on ultrafast timescales.

Excitons are bound electron-hole states that naturally form out-of-equilibrium in systems irradiated by light^1^. From the Materials Science perspective, they dominate the linear optical spectra of many emerging material systems such as two-dimensional solids^2^ and perovskites^3^, which has technologically enabled tunable light sources, photovoltaics, and quantum optical applications. At the Condensed Matter level, they are explored for their strong correlations inherently involving multi-electron states that cannot be described at the single-particle level^1^. Such physics paves way to many interesting phenomena involving signatures of excitons in measurements^4–7^, as well as more elaborate concepts like trions^8–10^, bi-excitons and multi-excitons^11–14^, and excitonic insulators^15–19^. Excitons are also of natural interest for higher-order optical nonlinearities where their contribution are not fully understood^20–24^.

Because excitons involve interactions between multiple electrons, and are also excited-states, their theoretical description can be highly cumbersome and numerically challenging. Common techniques for capturing excitonic features are based on many-body perturbation theory (e.g. GW + BSE^25^), or time-dependent density functional theory (TDDFT), as long as the latter employs long-range hybrid functionals with a portion of exact exchange^26^. The complexity of the theory creates a gap in our understanding of excitonic ultrafast dynamics – such costly numerical schemes usually cannot be directly applied in real-time and in realistic settings. Indeed, our understanding of exciton propagation is rather limited, with most recent works focusing on dissociation and dephasing^27,28^. Major open questions include how excitons initially form while interacting with lasers, how fast that happens (is it instantaneous?), and how they interact with other degrees of freedom such as band electrons, other excitons, and lattice excitations.

Here comes the work of Chen et al.^29^, who have employed real-time TDDFT with long-range interactions to directly study excitonic dynamics in a time-, space-, and momentum-resolved manner. Their work shows that exciton formation in resonant driving is not instantaneous. Instead, a 3-step mechanism is uncovered (see Fig. 1): (i) the laser first excites conduction band electrons (and valence band holes), (ii) electron-hole pairs bind to form an excitonic core within ~2.5 fs, (iii) the core evolves to its final form and population in well-defined states increases. The excitons then proceed to propagate while interacting with the laser and with each other, and interference is a dominant feature in the first tens of femtosecond prior to dephasing. Their work further suggests that even under strong laser fields a substantial portion of exciton population survives due to a continuous exciton excitation and sufficiently slow dissociation, which should affect connected processes such as high harmonic generation.

**Fig. 1:**
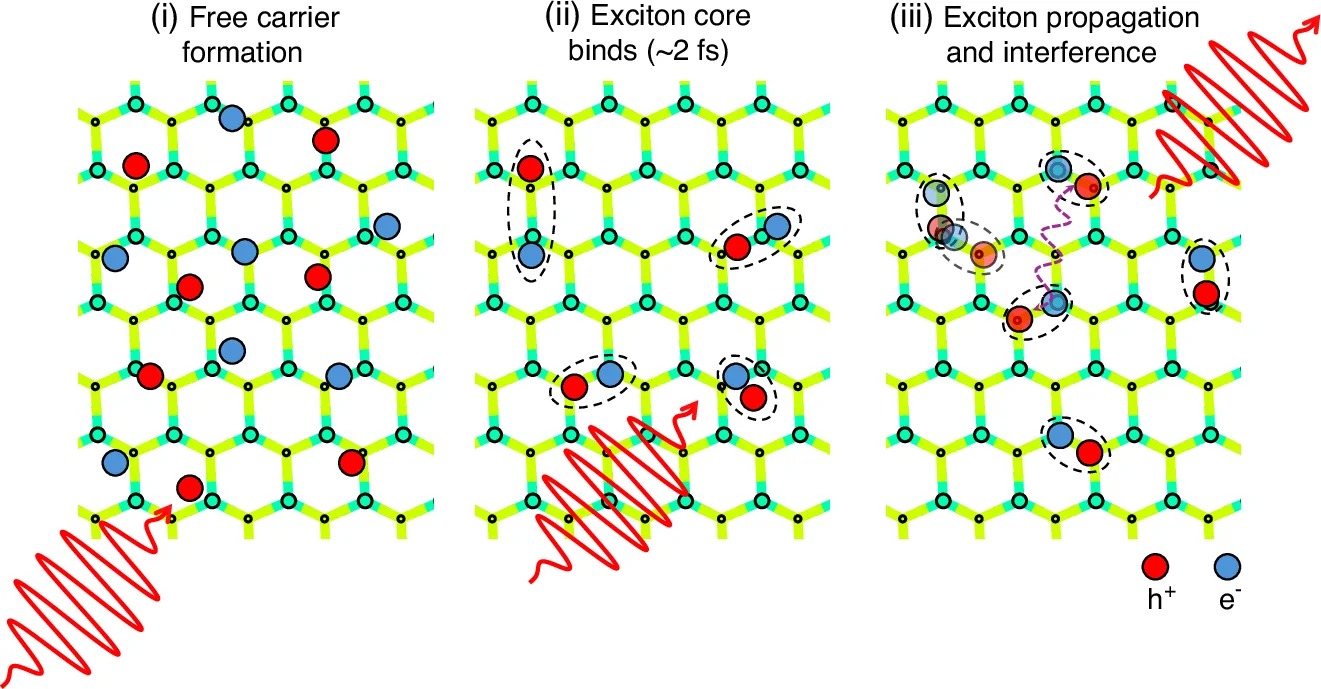
(left) Exciton formation under intense laser illumination. In the first femtosecond, free band carriers are formed (electrons in blue, holes in red). Subsequently (middle), electrons and holes begin to bind (while still interacting with the laser), generating an exciton core within ~2 femtoseconds (superpositions of excitonic states). (right) Excitons completely bind, generating the 1S exciton, and continue to propagate coherently (interacting with other excitons and interfering)

This physical mechanism proposes an intimate relationship between single-particle electron (and hole) states, and the many-body correlated states, whereby the two interchange and interact during the binding process and while interacting coherently with the laser field, connecting to similar physics in dissociation^27^. The impact of these results is potentially wide and applicable across fields – excitonic physics is essentially ubiquitous in out-of-equilibrium solid state as well as in molecular systems. Enhanced understanding of exciton dynamics in real time and space should therefore motivate novel optical schemes for manipulating exciton generation and tuning it in time, decoding excitonic interferences, and establishing physical connections across materials platforms. Applications of intense and tailored light^30^ for directly imaging exciton formation dynamics should also follow and open new research directions.
